# Treating wastewater contaminated with congo red (CR) dye using an optimized polyethersulfone/propolis (bee glue) PES/PRS ultrafiltration membrane

**DOI:** 10.1039/d5ra03565a

**Published:** 2025-07-07

**Authors:** Yusur Yahia, Khalid T. Rashid, Mohammed Ahmed Shehab, Adnan A. Abdul Razak, Maryam Y. Ghadhban, Munaf Al-lami, Mohammed A. Taher Al-Mayyahi, Mohammed A. Salih, Haidar Hasan Mohammed, Alhafadhi Mahmood

**Affiliations:** a Membrane Technology Research Unit, Chemical Engineering Department, University of Technology – Iraq Al Sinaa Street 52 10066 Baghdad Iraq che.22.24@grad.uotechnology.edu.iq Khalid.t.rashid@uotechnology.edu.iq adnan.a.alsalim@uotechnology.edu.iq maryam.y.ghadhban@uotechnology.edu.iq; b Polymers and Petrochemicals Engineering Department, Basrah University for Oil and Gas 61004 Basrah Iraq mohammed.ahmed@buog.edu.iq; c Department of Chemical Engineering and Petroleum Refining, Basrah University for Oil and Gas 61004 Basrah Iraq munaf.adnan@buog.edu.iq moh.may@buog.edu.iq mohammed.salih@buog.edu.iq haidar.alawaad@buog.edu.iq; d Thermodynamics and Mathematical Physics Unit, University of Mons 7000 Mons Belgium; e Department of Mechanical and Energetic, University of Dunaújváros Dunaújváros Hungary Alhafadhi@uniduna.hu; f Department of Mechanical Engineering, University of Sumer Rifai Iraq

## Abstract

The environment and human health are greatly suffering as a result of pollution. The textile sector is a significant generator of dyes, which are colored chemicals that are among the most significant water pollutants. In order to produce and optimize green mixed matrix membranes (MMMs) for the treatment of wastewater discharged from the textile industry, propolis (bee glue) was used as an additive to improve the performance of the PES membrane. This work utilized analysis of variance (ANOVA) and response surface methodology (RSM) to optimize the ultrafiltration (UF) process for the treatment of wastewater contaminated with Congo red. Specifically, the primary and interrelated effects of three preparation and operating parameters, additive content, transmembrane pressure, dye feed concentration on the membrane permeate flux, and CR dye rejection using the PES/PRS membrane were examined to optimize the UF process. Transmembrane pressure (1–5 bar), dye concentration (100–300 ppm), and PRS NP content (0–88 mg) were among the factors examined. Optimization results showed that PES/PRS MMMs were the most efficient, with a dye rejection rate of 99.8% and a flux of 48.44 kg m^−2^ h^−1^, at a PRS content of 64.43 mg, operating pressure of 5 bar and CR dye feed concentration of 210.08 ppm. Overall, this research not only shows that innovative PES/PRS UF membranes have the potential to treat wastewater containing dyes very well, but it also offers a useful comprehension of the dye-rejection process, which may direct the logical design of UF membranes. This work showed that the PES/PRS membrane would be a viable option for wastewater industrial applications in terms of its CR dye removal effectiveness under ideal operating circumstances.

## Abstract 2

The environment and human health are greatly suffering as a result of pollution.

## Graphical abstract

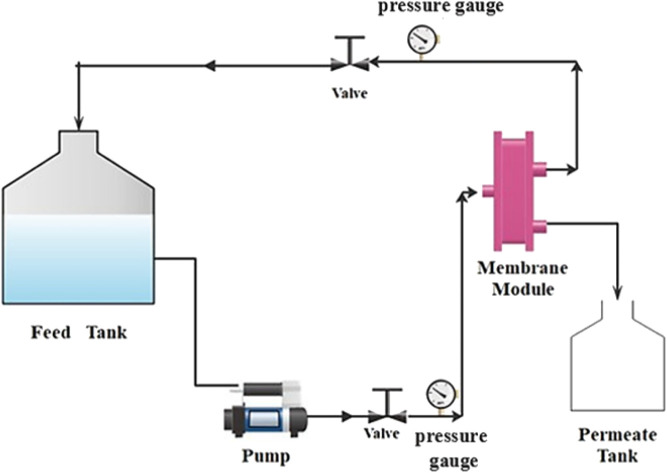

## 1. Introduction

The most pressing issue confronting humanity today is the scarcity of fresh water, brought about by increasing urbanization, industrialization, and population as well as climate change. Numerous organic and inorganic pollutants are introduced into water systems through human activities.^1,2^ Research has concentrated on appropriate alternative methods for obtaining freshwater from wastewater to address the issues related to water scarcity and stress in many areas. Superior water and wastewater treatment solutions have been proposed based on a number of cost-effective and multipurpose methods. Metal oxides, pigments, and dissolved organic debris have become significant concerns among contaminants because of their varied origins and possible harmful effects.^3–7^ Several techniques are available for disposing of aqueous solutions contaminated with dyes, which may be divided into three categories—physical, chemical, and biological—although none stand out above the rest. Other approaches are also being studied, including membrane technologies, since they are cost-effective and produce high yields. As a consequence, membrane technology has attracted widespread attention. Membranes function as a filter, retaining molecules larger than the membrane pores while allowing water to pass through. This technique is based on the separation of compounds on the basis of their particle size and charge. Over the previous decade, more than 60% of works have focused on nanoporous membrane production processes and their application in water purification.^8–12^ While these usually have several benefits, such as offering energy efficiency and selective separation, they also have certain drawbacks. The key limitation of the membrane filtering process is the membrane fouling phenomenon, whereby fouling occurs when impurities, such as organic matter, minerals, bacteria, or suspended particles, build up on the membrane surface, lowering its efficiency and raising maintenance costs. Fouling can also cause lower permeability, an increased operating pressure, and necessitate premature membrane replacement, and therefore higher cost.^13^

To overcome this problem, it is necessary to modify and optimize the properties and performance of the membranes. One promising way to improve membranes is by the use of additives. By adding inorganic nanoparticles of SiO_2_, Fe_3_O_4_, ZrO_2_, TiO_2_, and Al_2_O_3_ to the membrane,^14–18^ hydrophobic membranes can be transformed into very hydrophilic membranes. TiO_2_ is often used for membrane modification due to its photocatalytic and super-hydrophilic properties.^19,20^ TiO_2_ particles self-assemble on the membrane's surface *via* coordination bonds with the OH functional groups of the polymer, increasing the membrane's hydrophilicity in addition to its photocatalytic capabilities.^21^ Increasing the membrane's hydrophilicity is the main objective of membrane alteration as it will improve the membrane performance. Covalent linkages have been widely used to chemically modify hydrophilic polymers onto membrane surfaces, including zwitterionic polyelectrolyte, poly(ethylene glycol), poly(ethylene glycol) methyl ether methacrylate, poly(2-hydroxyethyl methacrylate), and poly(acrylic acid). The results show that hydrophilic membranes provide thick, hydrated layers that keep oil droplets from clogging the membrane surfaces and make oil removal easier during cleaning.^22,23^ A new and significant tactic to improve the membrane performance and support sustainability objectives is the use of green additives in polymer membranes. The use of green additives made from plant waste or sustainable resources has gained popularity in recent years. Green additives are eco-friendly substances, usually bio-based, biodegradable, and non-toxic, that can be added to polymer membranes to enhance a number of characteristics, including the membranes' mechanical strength, permeability and selectivity, hydrophilicity, surface qualities, fouling resistance (particularly biofouling), and thermal and chemical stability.^5,13,24^ Mixed matrix membranes (MMMs) involve the incorporation of a solid phase in a continuous polymer matrix. The application of these membranes is a good way to achieve contributory effects between the polymeric matrix and solid particles. Much work has been done to increase the efficiency of MMMs in order to achieve a high rejection rate, optimum permeate flow, and long-term processing viability. The best operating conditions for this process are determined by these requirements. For achieving suitable operating circumstances, a number of factors need to be considered, such as pressure, pH, solute feed concentration, and amount of additives. Ultrafiltration (UF), a membrane-based wastewater treatment method, is popular because of how well it removes impurities. However, a number of operational factors can have a significant impact on the membrane performance, and the treatment efficacy, membrane longevity, and cost-effectiveness can all be increased by optimizing these factors. The kind of membrane, the kind of wastewater, and the particular treatment objectives all affect the optimum operating parameters in membrane wastewater treatment. Nonetheless, the majority of membrane processes follow a few broad guidelines.^3,24^

Meenakshi *et al.*^25^ investigated how several operating parameters, such as the pH, feed concentration, cross-flow velocity, and transmembrane pressure, affect the removal of selenium from drinking water. Using response surface methodology-based optimal operating conditions, they found that their investigated module obtained a high degree of selenium separation (more than 98%). At an operating pressure of 14 bar, it could also continuously maintain a high flux of 140 LMH. Their work also demonstrated that when many factors and interactions affect the desired results, response surface methodology (RSM) is a useful tool to optimize the procedures. Indeed, many studies have shown that the response surface methodology (RSM) is a useful statistical and mathematical strategy to improve and optimize experimental processes impacted by many factors.^26,27^ Consequently, RSM assesses the links between variables and their impact on one or more quantifiable outcomes in addition to identifying the most advantageous value for each variable.

Propolis (also known as bee glue) has been utilized in a wide range of scientific and commercial applications, including encapsulation, microencapsulation, film casting, and the development of composite materials, thereby enhancing its potential for use in food-related applications. It can improve mechanical properties, as well as enhance the oxygen and moisture barrier functions, antioxidant capacity, and microbial resistance. Consequently, the incorporation of propolis into composite materials presents a promising avenue for future food packaging solutions. According to current findings, various industries could benefit from the production of propolis-based composites. In the context of polymer membrane applications, this study represents the first use of propolis. We aimed to utilize its previously highlighted favorable characteristics, such as efficient dispersion within the membrane matrix, hydrophobic nature, and capacity to enhance resistance to membrane fouling.^28,29^

Specifically, we aimed to investigate the use of a green propolis (bee glue) mixed matrix membrane to modify the ultrafiltration process. Response surface methodology (RSM) and variance analysis (ANOVA) were used to optimize the UF preparation and operating parameters. Here, the research employed Design-Expert® software to investigate several operational parameters, including the transmembrane pressure (1–5) bar, CR dye concentration (100–300 ppm), and PRS NPs content (0–88 mg), in order to optimize them. The ranges of the preparation and operating variables were chosen based on the experimental setup's capacity, cost concerns, and membrane working restrictions. The purpose of these tests was to ascertain how they affect the CR dye rejection percentage and membrane permeate flux. The research also looked at how the operating parameters can work together to maximize the performance of PES/PRS-based membranes.

## 2. Experimental section

### 2.1 Materials

Polyethersulfone (PES), a basic polymer with a molecular weight of 30 000 g mol^−1^, was provided by S.A.P in Belgium for the manufacturing of the membrane. Propolis was extracted from nearby bee hives. Di-methyl-formamide (DMF) solvent was bought from Sigma-Aldrich, Germany, while Congo red dye, C_32_H_22_N_6_Na_2_O_6_S_2_, with a molecular weight of 696.66 g mol^−1^, was purchased from BDH Company (Staines, UK).

### 2.2 Preparation of the PES/PRS membranes

The mixed matrix membranes (PES/PRS) were synthesized using a process of non-induced phase separation, by controlling the rate at which solvents and nonsolvents (in coagulation water baths) exchange in the phase-inversion process, as outlined in our previous work.^30^ All the flat sheet membranes were fabricated using the phase-inversion technique. Prior to the manufacturing of the casting solution, the PES polymer was subjected to drying at 50 °C for 10 h in an oven to eliminate any moisture present. A dope polymer solution was produced by combining the solvent dimethylformamide with PES powder polymer in a beaker at 25 °C. PRS was introduced in separate increments to the casting solution, which was agitated for 2 h using a magnetic stirrer until homogenization was achieved. The solution was then placed in an ultrasonic bath for half an hour, followed by a 1 h incubation in an oven at 40 °C to facilitate the release of any bubbles prior to the casting process. The resulting homogeneous solution was subsequently applied onto a flat glass using a casting machine equipped with a 0.2 μm air gap casting knife. Upon completion of the pouring process and the subsequent application of the knife, the flat glass was submerged in a bath of distilled water maintained at 25 °C. The casting solution underwent phase inversion to achieve a solid state and was maintained in distilled water for 48 h to guarantee complete phase inversion. The compositions of the casting solutions for the various membranes are listed in Table 1.

**Table 1:** UF membrane compositions

Sample	PES (wt%)	PRS (mg)	DMF (wt%)
YP0	20	0	80
YP1	20	12.5	80
YP2	20	37.5	80
YP3	20	62.5	80
YP4	20	87.5	80

### 2.3 Membrane performance

**Fig. 1:**
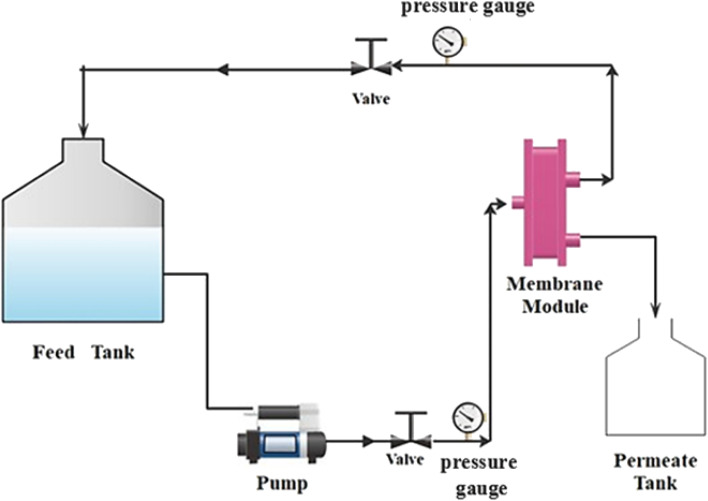
Process flow schematic for the ultrafiltration setup at the laboratory size.

A cross-flow filtering system, shown in Fig. 1, with an effective area of 14 cm^2^ was used to evaluate the membrane performance at room temperature. Analysis of the membrane penetration was conducted using a pure water flux (PWF) and 100, 200 and 300 ppm solutions. The permeate flow (*F*) was determined using eqn (1).1
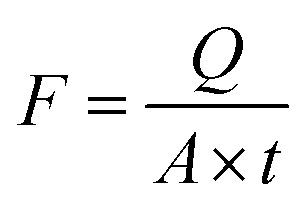
where *Q* denotes the permeate of the collected volume (L) of the water/wastewater feed, *A* denotes the membrane area (m^2^), and *t* denotes the filtration duration (h).^31^

Eqn (2) was used to determine the optimum membrane parameters and find the CR red dye retention (*R*).2
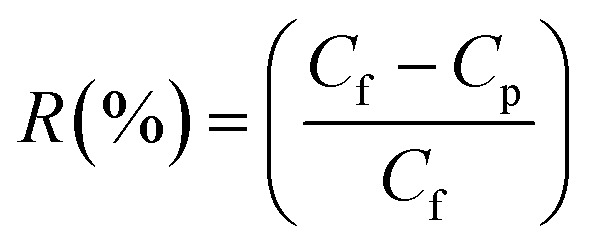
where *R* is the membrane retention, *C*_f_ is the permeate dye concentration and *C*_p_ is the CR dye concentration in the feed streams in mg L^−1^.^31,32^

### 2.4 Fourier transform infrared (FTIR) spectroscopy analysis

**Fig. 2:**
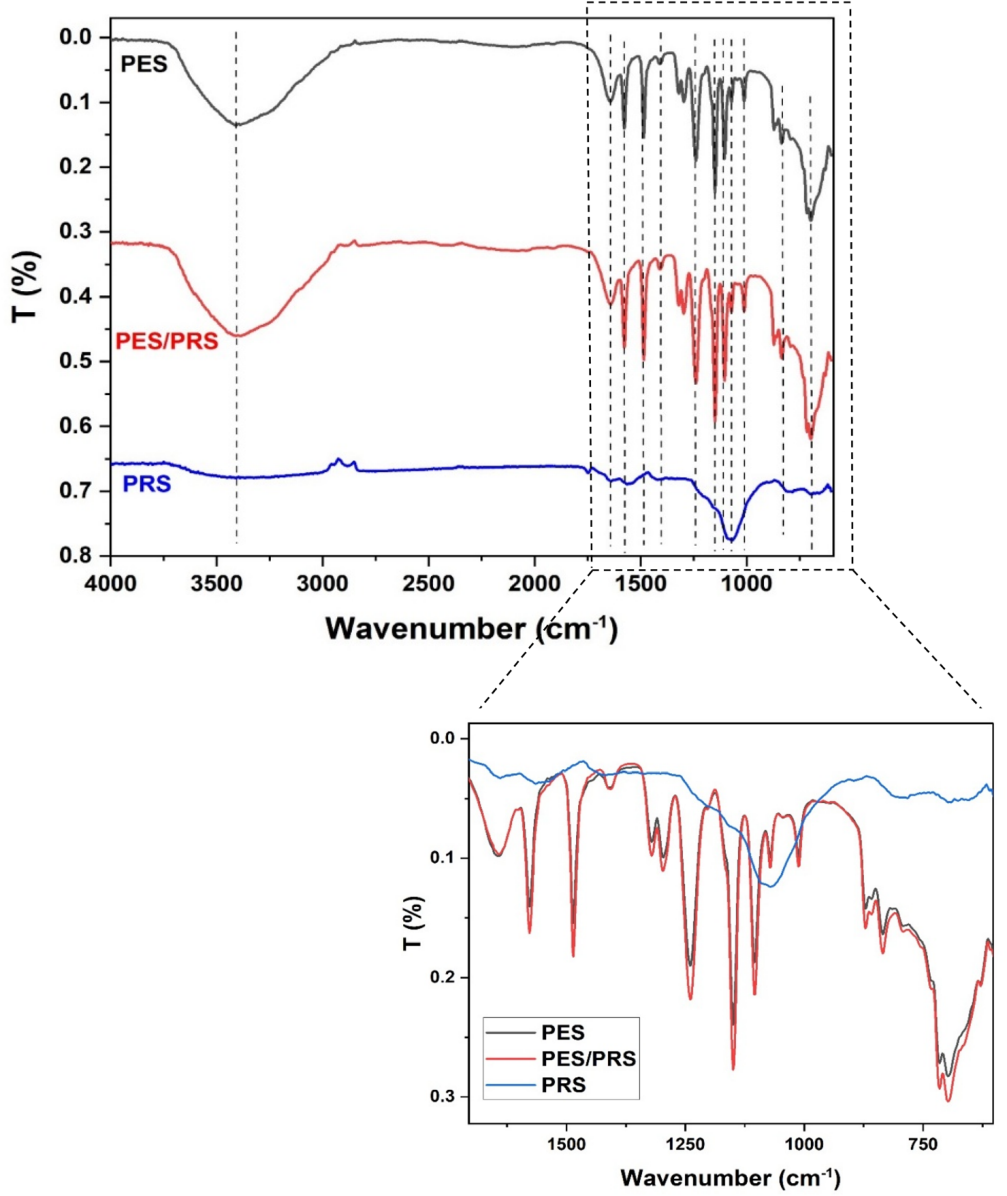
Fourier transform infrared (FTIR) spectra of the synthesized PES/PRS membrane and propolis nanoparticles (PRS NPs).

Fig. 2 illustrates the FTIR analyses of PES, PRS, and PES/PRS. The chemical structure of PES comprised three significant functional groups: benzene, ether, and sulfone. The presence of benzene rings could be expected to show three peaks within the range of 1600–1400 cm^−1^. In the PES spectrum, these peaks were identified at 1577, 1485, and 1409 cm^−1^. The ether function was confirmed by the detection of its characteristic peaks at 1321 and 1298 cm^−1^. The two characteristic peaks associated with the sulfone group (–SO_2_) were observed at 1149.5 and 1105 cm^−1^. The vibration bands of the C–S bond were observed at 696 and 715.5 cm^−1^, while the signals at 835 and 871 cm^−1^ corresponded to vibrations of the C–H bond. Bee glue (propolis) has bands in the same region of PES; due to that, after adding the propolis to the PES membrane structure, we could not observe new bands or at least band shifting. However, the increased band intensity observed in the FTIR spectrum of the propolis-modified PES (PES/PRS) membranes indicated the integration of propolis within the membrane structure, leading to alterations in the membrane's chemical composition and functional characteristics.^33–36^

### 2.5 Experimental design

Concerning membrane manufacturing, it is essential to optimize the conditions for experimentation to reduce resources, such as energy, and the time required, while enhancing membrane performance. In the majority of investigations, optimization studies have employed the one-factor-at-a-time methodology. However, this methodology is labor-intensive and necessitates numerous experiments.^37^ A frequently utilized alternative optimization technique is response surface methodology (RSM).^38^

Response surface methodology is a mathematical and statistical approach utilized for experimental design. The aim is to enhance the response affected by multiple independent variables.^39^ RMS was also designed to measure the influence of operational variables and their impact on response parameters.^40^

RSM offers numerous advantages over the commonly used traditional method, including enhanced speed, reliability, and more informative insights, significantly lowering the total number of experimental runs needed. This efficiency accelerates experimental work and lowers costs.^41^ Moreover, RSM enables the identification of the optimal factors required to achieve the best outcomes.^42^

Herein, the evaluations were undertaken utilizing Design-Expert® software. This work aimed to optimize several operational parameters through investigating the PRS NPs concentration (0–88 mg), concentration of feed dye input (100–300 ppm) and transmembrane pressure (1–5 bar), to evaluate their effects on membrane flux and the % dye retention. Furthermore, this research investigated the synergistic impact of the operational factors on the results.

To determine the impact of independent variables on dependent parameters in a regression study, researchers typically utilize analysis of variance (ANOVA), a set of statistical models for examining the variances between several variables. Table 2 presents the experimental data and variable symbols used herein. A series of experiments were conducted applying the ultrafiltration membrane process, with each test altering a single variable to determine the necessary operating conditions while maintaining the other variables constant.

**Table 2:** Parameter codes and factor levels

Parameters	Coded	Unite	Low level	High level
Dye conc.	A	ppm	100	300
Add. conc.	B	mg	0	88
Press	C	bar	1	5

RSM was utilized as an optimization approach to study how various parameters (predictors) affected the process of the membrane flux and dye rejection percentage. Table 3 shows the detailed historical data related to the 20 runs conducted. The data points provided the critical design parameters for modeling and optimizing the permeate flux and dye retention.

**Table 3:** Response and experimental data points

Runs	*A*: initial dye con. (ppm)	*B*: additive loading (mg)	*C*: pressure (bar)	Rejection%	Flux (kg m^−2^ h^−1^)
1	200.00	44.00	3.00	99.8	39.2
2	100.00	88.00	1.00	95.6	33
3	100.00	0.00	5.00	83	23.66
4	200.00	44.00	5.00	99.6	44.5
5	100.00	88.00	5.00	93	53
6	100.00	0.00	1.00	88.6	13.34
7	200.00	88.00	3.00	98	39.2
8	100.00	44.00	3.00	97.6	41.3
9	200.00	44.00	3.00	99.8	39.2
10	300.00	44.00	3.00	99.9	37
11	300.00	88.00	1.00	96.7	43
12	200.00	44.00	3.00	99.8	39.2
13	300.00	88.00	5.00	97.8	41.6
14	200.00	44.00	3.00	99.8	39.2
15	200.00	0.00	3.00	89	12.4
16	200.00	44.00	1.00	99.6	38.5
17	300.00	0.00	5.00	90	8.2
18	200.00	44.00	3.00	99.8	39.2
19	300.00	0.00	1.00	90.2	12.5

## 3. Results and discussion

### 3.1 Membrane filtration cross-flow evaluation

Every PES/PRS membrane outperformed the base membrane in terms of pure water flux (PWF) and dye rejection. Even with a little amount of PRS nanoparticles, addition of the green NP additives had a major impact on the flow characteristics. The water flow rose from 20 (kg m^−2^ h^−1^) for the original membrane to 34 (kg m^−2^ h^−1^) with the addition of 12.5 mg of PRS NPs, according to the data. When PRS NPs were added, the PWF increased significantly; for YP3, the highest value was 63.28 kg m^−2^. As previously shown, the rise in PWF was in line with the manufactured membrane's hydrophilicity, porosity, and pore size measurements. It is worth noting when 87.5 mg of PRS NPs was added, the pure water flux dropped slightly. The PWF decreased as a consequence of pore constriction brought on by the increased concentration of PRS NPs. The performance of the membranes was weakened as a result of the agglomeration phenomena brought on by the casting solution's increased PRS concentration.^3^

### 3.2 Regression model equations and ANOVA analysis

ANOVA analysis was conducted utilizing Design-Expert®. The findings of the analysis of the (ANOVA) variance for the permeate flux and % dye rejection, considering the concentration of PRS NPs in mg, are shown in Tables 4 and 5, showing the transmembrane pressure measurements and dye concentrations. In the design layout, each predictor variable is associated with a specific *p*-value in the ANOVA results table. The findings were analyzed to derive a mathematical form. Equations for the regression model concerning the % rejection and permeate flux are presented as eqn (3) and (4) below, based on the actual variables related to the membranes.

3 *F* (kg m^−2^ h^−1^) = +10.41956 + 0.021675 *A* + 0.78010 *B* + 1.52586 *C* + 4.23295 10^−4^ AB − 0.022512 AC + 0.017869 BC + 1.30928 10^−5^*A*^2^ − 6.82803 10^−3^*B*^2^ + 0.62023 *C*^2^

4 *R* (%) = +85.12001 + 0.050865 *A* + 0.38151 *B* − 1.25553 *C* − 7.67045 10^−5^ AB + 5.68750 10^−3^ AC + 6.10795 10^−3^ BC − 1.19381 10^−4^*A*^2^ − 3.32842 10^−3^*B*^2^ − 0.085954 *C*^2^

where *F* is the flux (kg m^−2^ h^−1^), *A* is the dye concentration (ppm), *B* is the PRS NPs mass feed (mg), and *C* is the transmembrane pressure (bar) and *R*% is the % rejection.

**Table 4:** Membrane permeate flux analyzed using ANOVA

Source	Sum of squares	df	Mean square	*F* Value	*p*-Value prob > *F*	
Model	2973.24	9	330.36	445.62	<0.0001	Significant
*A*–*A*	48.40	1	48.40	65.29	<0.0001	
*B*–*B*	1951.61	1	1951.61	2632.50	<0.0001	
*C*–*C*	93.76	1	93.76	126.47	<0.0001	
AB	27.75	1	27.75	37.43	0.0002	
AC	162.18	1	162.18	218.76	<0.0001	
BC	19.78	1	19.78	26.68	0.0006	
*A* ^2^	0.047	1	0.047	0.063	0.8072	
*B* ^2^	477.47	1	477.47	644.05	<0.0001	
*C* ^2^	16.82	1	16.82	22.69	0.0010	
Residual	6.67	9	0.74			
Lack of fit	6.67	5	1.33			
Pure error	0.000	4	0.000			
Cor. total	2979.92	18				
Std. dev.	0.86		*R*-Squared		0.9978	
Mean	33.54		Adj *R*-squared		0.9955	
C.V. %	2.57		Pred *R*-squared		0.9524	
Press	141.73		Adeq precision		71.224	

**Table 5:** Rejection analysis *via* ANOVA

Source	Sum of squares	df	Mean square	*F* Value	*p*-Value prob > *F*	
Model	470.01	9	52.22	156.16	<0.0001	Significant
*A*–*A*	28.22	1	28.22	84.39	<0.0001	
*B*–*B*	162.41	1	162.41	485,62	<0.0001	
*C*–*C*	5.33	1	5.33	15.93	0.0031	
AB	0.91	1	0.91	2.72	0.1332	
AC	10.35	1	10.35	30.95	0.0004	
BC	2.31	1	2.31	6.91	0.0274	
*A* ^2^	3.89	1	3.89	11.64	0.0077	
*B* ^2^	113.46	1	113.46	339.25	<0.0001	
*C* ^2^	0.32	1	0.32	0.97	0.3514	
Residual	3.01	9	0.33			
Lack of fit	3.01	5	0.60			
Pure error	0.000	4	0.000			
Cor. total	473.02	18				
Std. dev.	0.58		*R*-Squared		0.9936	
Mean	95.66		Adj *R*-squared		0.9873	
C.V. %	0.60		Pred *R*-squared		0.9386	
Press	29.04		Adeq precision		40.330	

Positive factor terms in eqn (3) indicate a positive impact on the flow, whereas negative factor terms indicate a negative influence. A positive sign in the factor term in eqn (4) indicates a favorable effect on the rejection, while a negative sign indicates a detrimental effect. The *R*-squared correlation coefficients for the permeate flux and rejection percentage models were 99.78% and 99.36%, respectively, and 99.55% and 98.73%, respectively, for the modified *R*-squared values. The results obtained for the *R*-squared values gave a satisfactory indication of the consistency of the tested results with the optimization values.

The data indicate that the permeate flux and retention percentage models were statistically valid and could accurately predict the performance of the UF membranes. The permeate flow and rejection percentage models' regression analysis showed that the model's correctness was highly precise and fitted the given data well.^43^

The anticipated *R*-squared correlation coefficient (predicted) values closely matched the (adjusted) correlation coefficient *R*-squared values for each model, as shown in Tables 4 and 5. Thus, both models included significant terms.

**Fig. 3:**
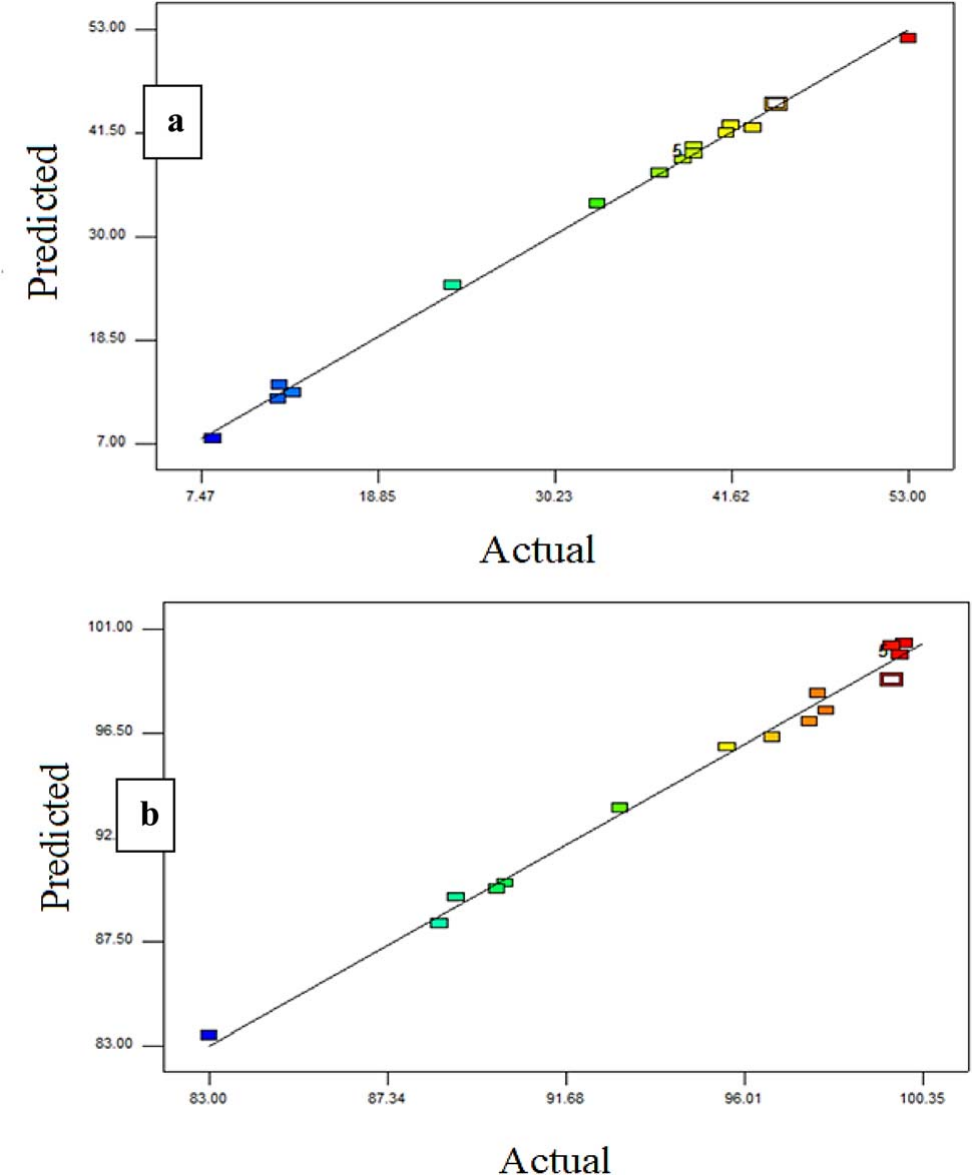
Actual and forecasted outcomes for the (a) permeate flux % and (b) dye rejection %.

A linear regression algorithm was employed in Design-Expert® software for estimating the flux and rejection percentage. Fig. 3 illustrates the actual and anticipated outcomes for the permeate flux and dye rejection percentages. The actual and predicted levels of membrane flux and the rejection percentages exhibited substantial mathematical concordance, as illustrated in Fig. 3a and b. The previous result and the elevated *R*-squared values (99.78% and 99.36% for the permeate flux and retention percentages, respectively) of the PES/PRS flat sheet membrane indicated the model's substantial potential for prediction and optimizing the permeate flux and % rejection.^44^

### 3.3 Main effects plot

**Fig. 4:**
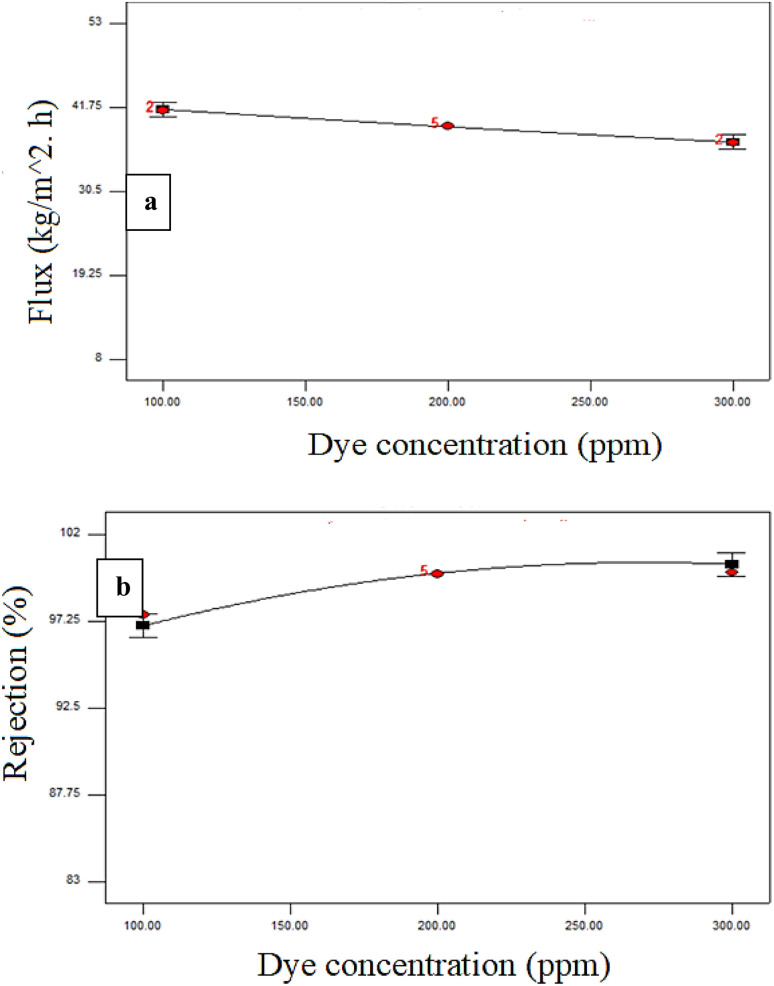
Dye concentration (ppm) effect on the (a) permeate flux and (b) dye rejection at a constant pressure of 3 bar and additive loading of 44 mg.

**Fig. 5:**
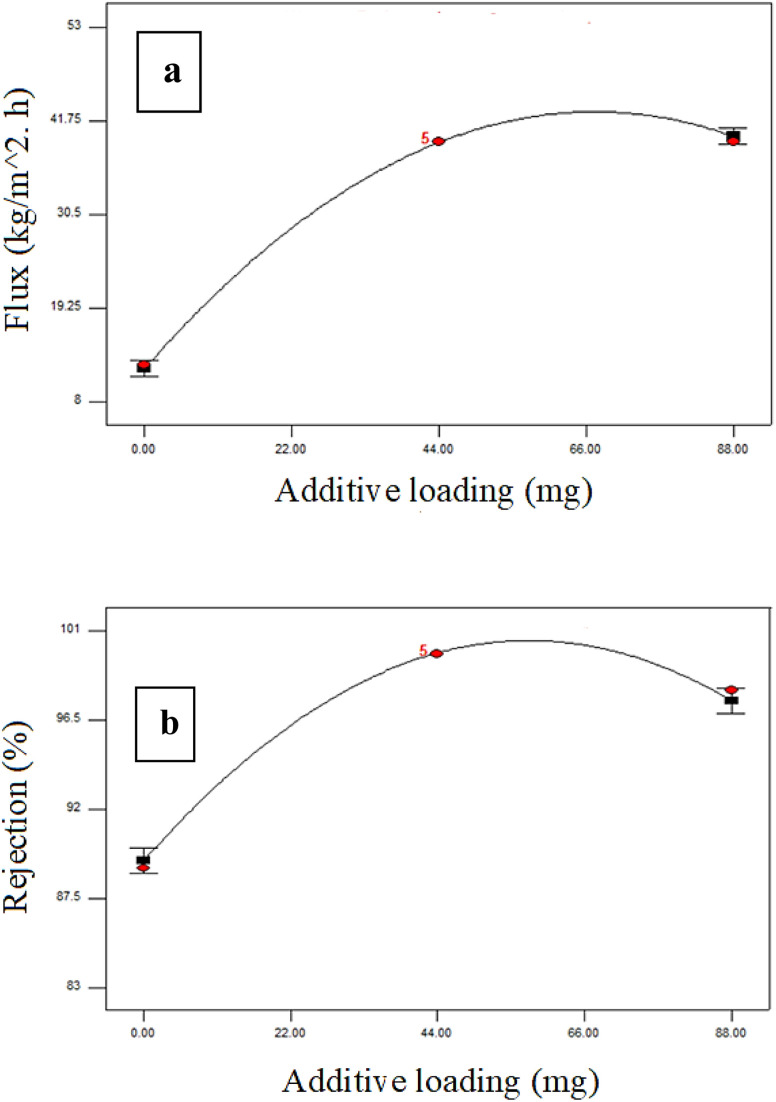
Additive loading effects on the (a) membrane flux and (b) dye rejection % at a constant initial concentration of 200 ppm and pressure of 3 bar.

**Fig. 6:**
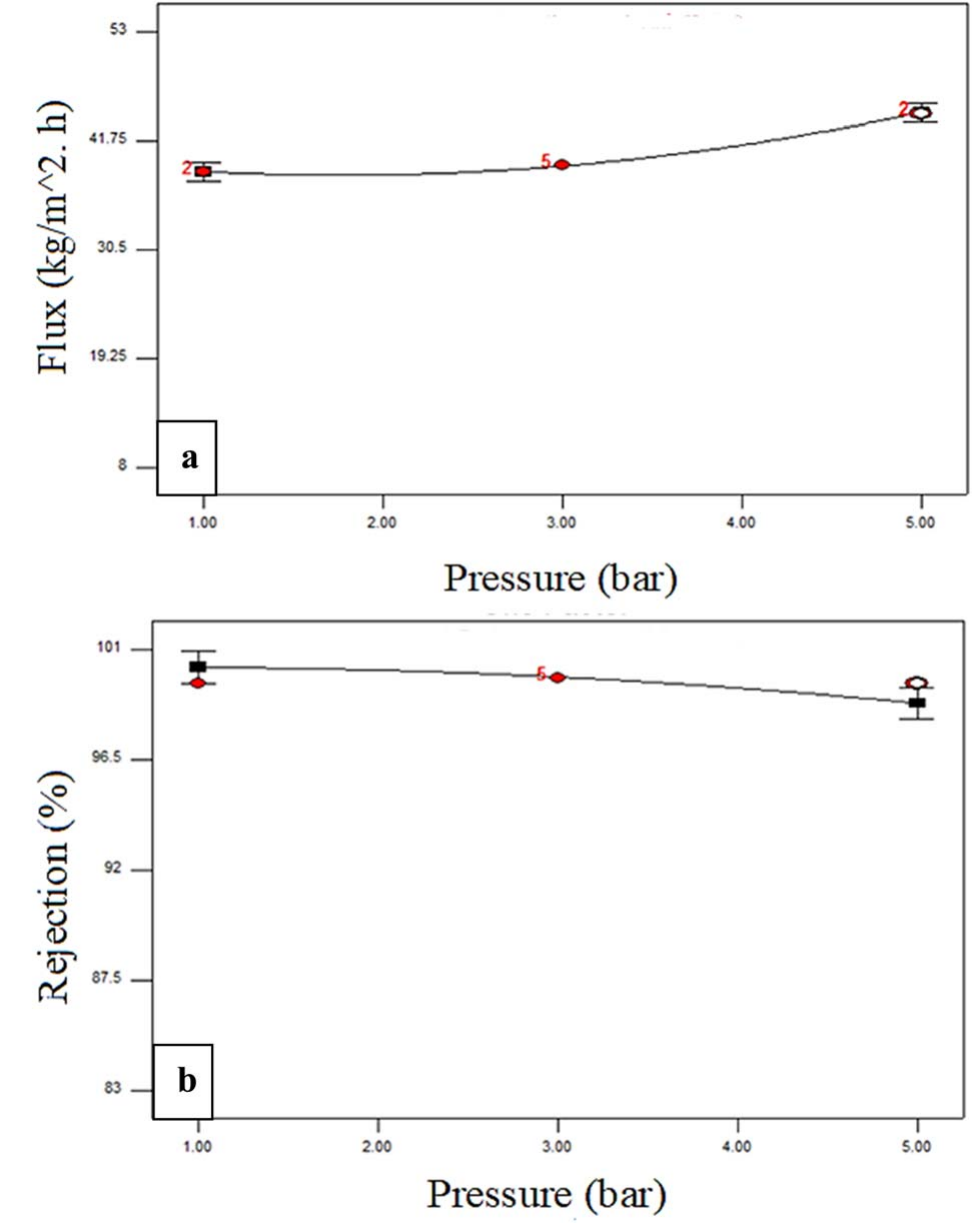
Effect of operating pressure on the (a) dye rejection% and (b) flux at a constant initial concentration of 200 ppm and additive loading of 44 mg.

Fig. 4–6 illustrate the analysis conducted by Design-Expert® software regarding the influence of the experimental factors and their interactions. One variable was altered each time to examine the influence of the factor, while the others were kept constant. A zero slope when varying the component from low to high levels implies that the variable has a negligible impact. A slope of one signifies that the variable exerts a positive influence on the response.

Fig. 4 illustrates the relationship between the percentage dye rejection and permeate flux for membranes with an additive concentration of 44 mg and a constant transmembrane pressure of 3.0 bar. The flux decreased with the increase in dye concentration (100–300 ppm). The aggregation of dye particles on the membrane reduced the flux, as shown in Fig. 4a.^45,46^

The rejection percentage for the membrane increased as the dye concentrations increased from 100 to 300 ppm. The existence of dye particles on the surface of the membrane could form a thin, dense layer, increasing the overall rejection rate, see Fig. 4b.^47^

Fig. 5 illustrates the influence of the additive concentration on the membrane flux and dye rejection percentage at a fixed dye content of 200 ppm and pressure of 3.0 bar. Fig. 5a illustrates that the flux was enhanced with a rise in additive content to 44 mg. Increasing the quantity of PRS NPs in the PES membrane polymeric solution improved the membrane's porosity, average pore size, and hydrophilicity.^48^ Conversely, increasing the concentration of additives to 88 mg diminished the flux due to the agglomeration of PRS nanoparticles on the membrane surfaces, leading to decreased porosity, hydrophilicity and also pore size effects; the permeate flux decrease was thought to be caused by this agglomeration.

Upon assessing the rejection percentage of the membranes, an increase in rejection was noted as the additive concentration rose. This inclination may be associated with the hydrophilic PRS NPs enhancing the membrane surface hydrophilicity.^49^ Moreover, as the concentration of PRS NPs increased to 88 mg, the dye rejection diminished, as illustrated in Fig. 5b. The reduction in dye rejection may be associated with increasing levels of PRS aggregation. This results in the formation of defects during the membrane production process, thereby negatively impacting the membrane's rejection performance.^50^

Fig. 6 illustrates the impact of varying the operating pressure (1 to 5 bar) on the permeate flux and dye rejection percentage of the membranes, while maintaining an unchanged dye concentration of 200 ppm and an additive content of 44 mg. The flux was amplified when the pressure was escalated from 1 to 5 bar. In the diffusion and solution model, flow is inextricably linked to pressure difference across a membrane.^51,52^ Nevertheless, the dye membrane rejection percentage diminished only a little as the pressure increased from 1 to 5 bar.

### 3.4 Effect of PRS additives concentration, dye concentration and pressure on the flux and rejection %

**Fig. 7:**
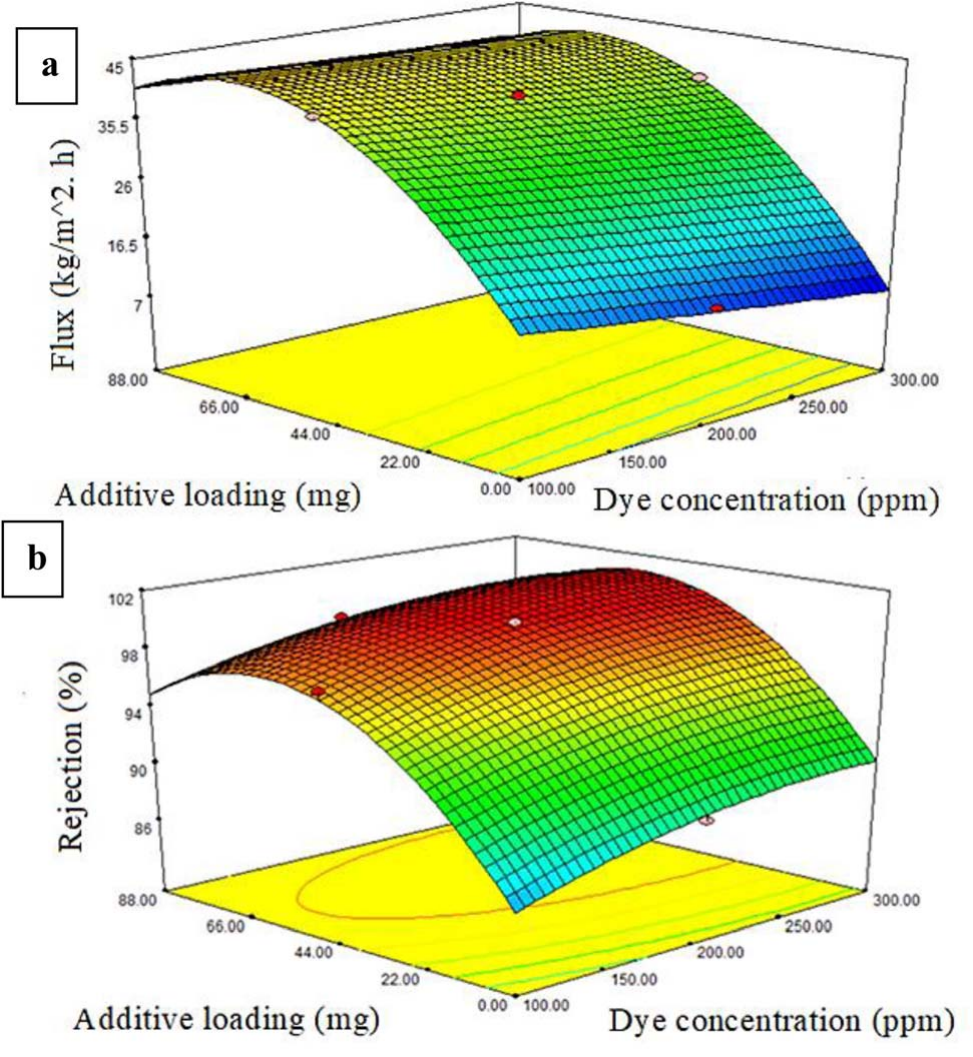
Impact of the PRS additive loading and dye concentration on the (a) membrane flux and (b) % dye rejection at 3 bar pressure.

**Fig. 8:**
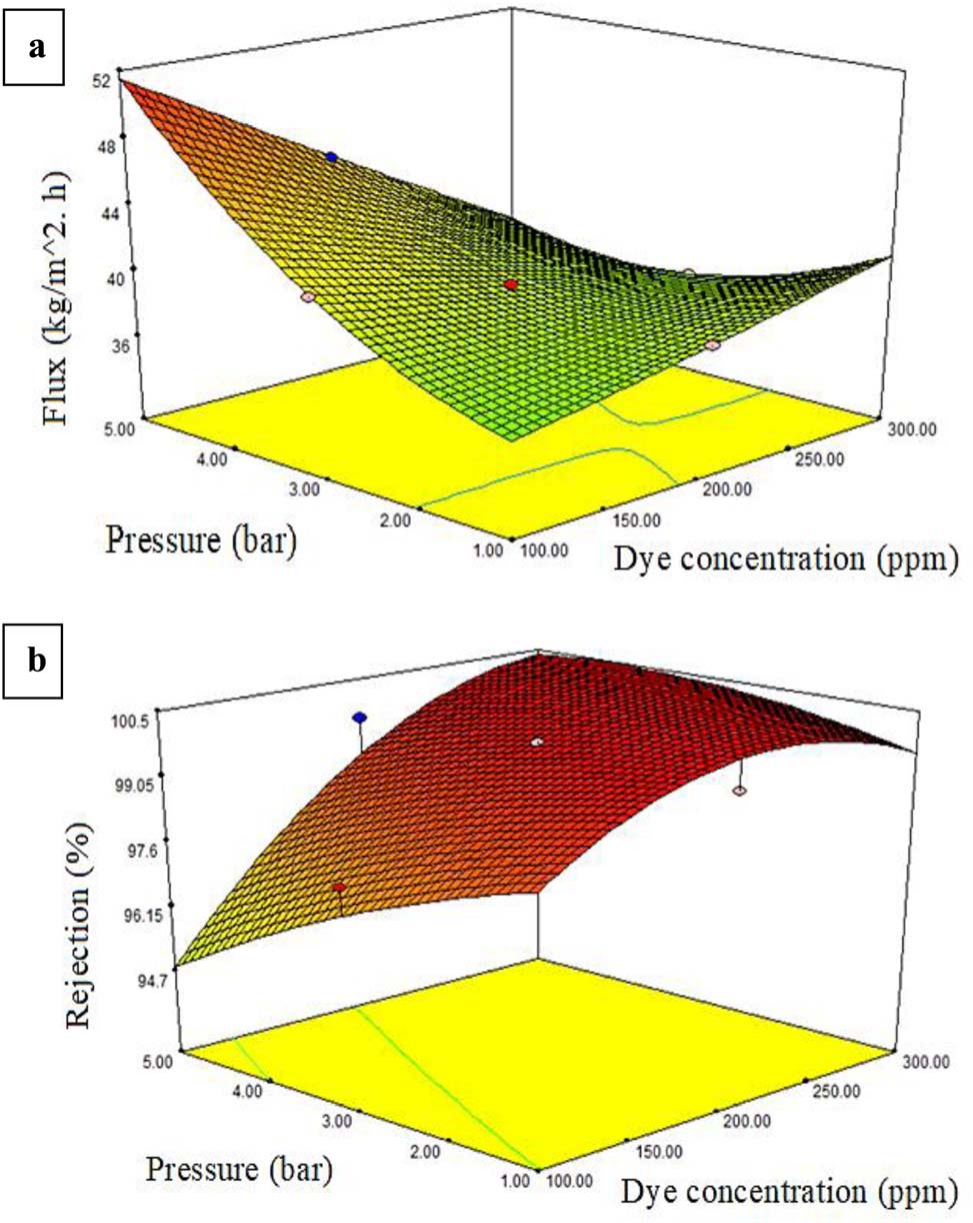
Effect of dye concentration and pressure on the (a) flux and (b) dye rejection (%) at an additive loading of 44 mg.

**Fig. 9:**
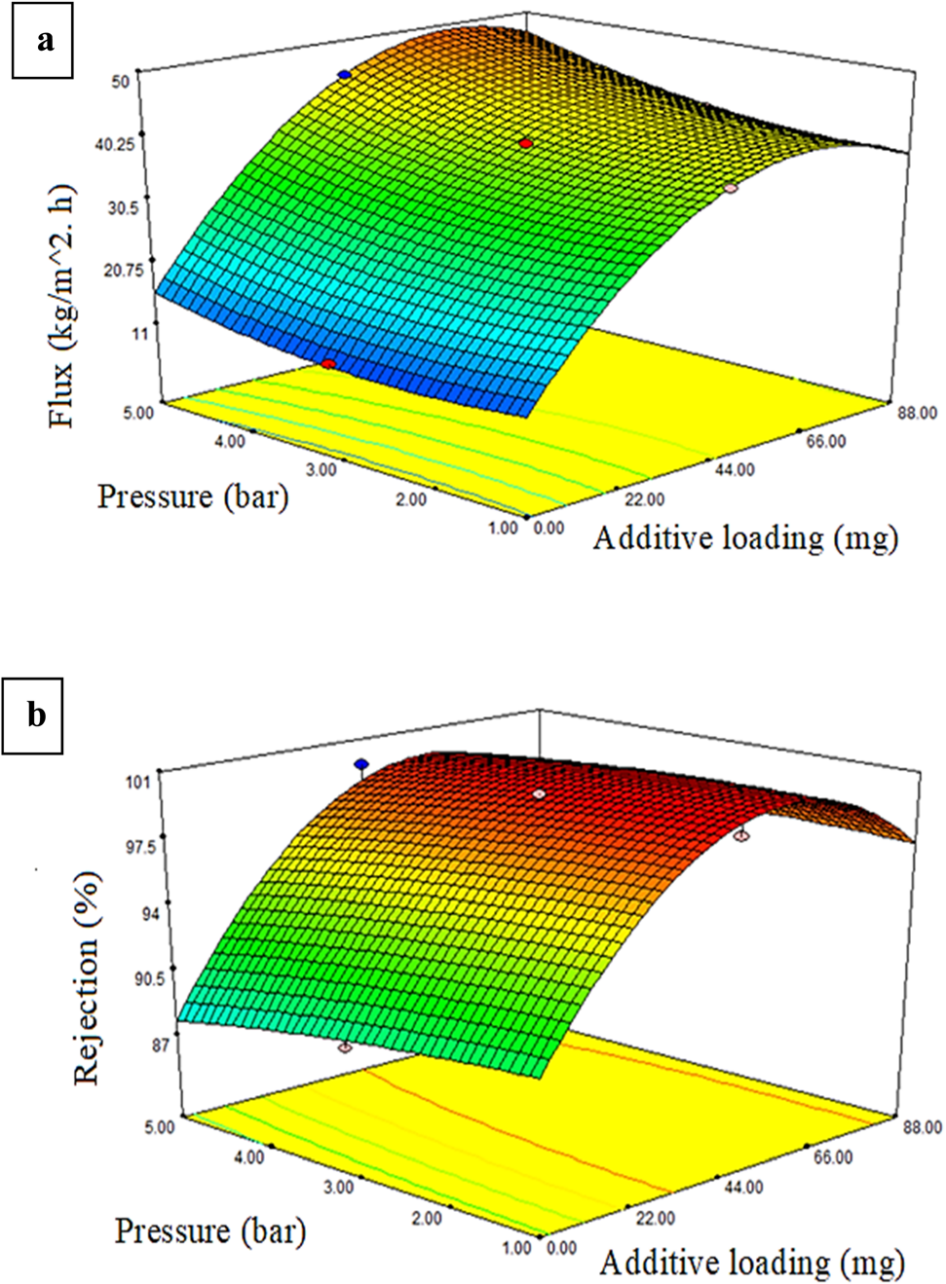
Pressure and additives effects of the (a) flux and (b) % dye rejection at a starting concentration of 200 ppm.

The dye response flux and dye percentage rejection can be described as a three-dimensional solid. Fig. 7–9 illustrate the permeate flux and dye rejection percentage in relation to the two variables. The main objective of the plot of the response surface was to identify the optimal operational parameters, specifically the dye concentration, additive concentration (mg), and pressure, which would lead to the maximum permeate flux and dye rejection percentage.

Fig. 7a presents a plot of the response surface demonstrating the influence of the dye content and PRS content on the dye/PWF under fixed pressure conditions. The dye/PWF response indicated a decrease in dye concentration, while an increase in additive concentration to 62.5 mg led to an enhancement in the flux. The highest flux observed was 63.28 kg m^−2^ h^−1^ with a PRS content of 62.5 mg of PRS NPs. Elevating the content of PRS NPs in the PES polymeric solution improved the hydrophilicity, average pore size, and porosity of the membrane. Conversely, increasing the concentration of additives to 87.5 mg led to a reduction in the flux, attributed to the agglomeration of PRS additives on the surface of PES membranes, which resulted in a decrease in the porosity, pore size, and hydrophilicity.^53^

The dye rejection percentage response demonstrated that the increase in additive content to 62.5 mg, coupled with a decrease in dye concentration, led to a reduced rejection percentage at a fixed pressure, as shown in Fig. 7b. The elliptical contour plots demonstrated there was an interaction between the dye feed amount and PRS additive content.

The response surface plot, shown in Fig. 8a, demonstrates how the dye concentration and pressure effected the permeate flux. The permeate flow decreased as the dye concentration rose and the pressure decreased. Reducing the pressure from 5 to 1 bar significantly reduced the flux, while increasing the amount of dye had a smaller impact compared to the pressure and only led to a small decline in the flux.

The relationships between the pressure, dye content, and dye rejection percentage are illustrated in Fig. 8b as a response surface plot. A negative effect on rejection was shown with the slight decrease in membrane rejection resulting from raising the pressure.^54^ Hence, at higher pressures, the rejection rate dropped, but the rejection percentage went up as the dye content went up. A thin, compact layer could be formed by the dye molecules on the surface of the membrane, increasing the total rejection rate and leading to an improvement in rejection. Still, the contour plots' structure of parallel straight lines was responsible for the lack of interaction between these components.

Fig. 9a presents a three-dimensional representation of the response surface, demonstrating the impact on the dye/water flux from the interplay between the pressure and additive content. At a fixed dye quantity of 200 ppm, an increase in the PRS additive amount to 44 mg resulted in a greater enhancement of the dye/water flux compared to the effect of pressure. The rise in flux was generally related to combined effects from elevating the PRS NPs quantity in the PES polymer solution, enhancing the hydrophilicity, growing the membrane's mean pore size and porosity, and promoting the pressure. Consequently, the flux increased because of the higher shear stress on the membrane surface.^55^

Fig. 9b illustrates the relationship between the additive amount and pressure on the dye rejection percentage while maintaining a constant dye content. Elevating the additive quantity to 44 mg improved the dye rejection rate, whereas the increase in pressure resulted in decreased rejection rates. Increasing the additive quantity to 44 mg improved the rejection rate, even in high-pressure conditions. The contour plots' elliptical form suggested there was a strong relationship between the effects of the operating pressure and the PRS NPs additive quantity.

### 3.5 Validity and optimization of the UF procedure

**Fig. 10:**
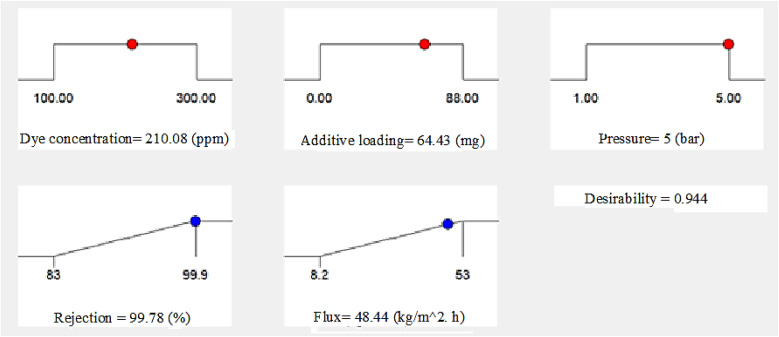
Desirability ramps for numerical optimization of the three chosen parameters.

One of the most well-known approaches to optimize different reaction processes in the fields of applied science and engineering is the desired function analysis. This technique takes a single value on a scale from one to zero and uses it to integrate all the responses concerning an individual desirability. Optimal operating conditions are defined using values closer to one, since this represents the optimum case situation. However a value close to zero means that at least some of the responses are not within the target range.^56^ We used Design-Expert® software to determine the desirability function for the two current responses, namely the flux and dye percentage rejection, as shown in Fig. 10. This process integrated the individual's desirability into a single value. The accompanying figure shows the optimum operating parameters that were investigated, which were the dye concentration, pressure, and additive concentration. It was expected that these three factors would considerably increase the dye/water flux and rejection factor. In addition, the model was used to confirm the experimental results for the flux and % dye rejection under the recommended ideal operating parameters. According to Table 6, there was a discrepancy of 2.086% between the experimental values (47.45 kg m^−2^ h^−1^) and the predicted flux (48.44 kg m^−2^ h^−1^) for the membranes. Furthermore, the experimental value (98%) was 1.816% off from the predicted rejection % (99.78%). The generated RSM model was thus shown to be significant and applicable, as shown by the modest percentage error.

**Table 6:** Pred. *versus* exp. values for the dye rejection percentage and flux under ideal circumstances

Add. conc. (mg)	Dye conc. (ppm)	Press (bar)	*R* (%) Pred.	*R* (%) Exp.	Error %	*F* (kg m^−2^ h^−1^) Pred.	*F* (kg m^−2^ h^−1^) Exp.	Error %
64.43	210.08	5	99.78	98	1.816	48.44	47.45	2.086

## 4. Conclusion

In order to remediate wastewater tainted with Congo red dye, this work examined the effects of embedding propolis (bee glue) (PRS) nanoparticles in PES mixed matrix membranes. Pristine and modified membranes were extensively investigated in terms of their contamination rejection percentage and permeate flux using Congo red dye solution. Analysis of variance (ANOVA) and response surface methodology (RSM) were used as statistical and mathematical techniques to improve the process' efficacy on a larger scale. In the ultrafiltration process, the effects of the preparation and operating parameters on the permeate flow and Congo red dye rejection percentage of the PES/PRS mixed matrix membranes were optimized. The PRS NPs weight percentage content, CR dye concentration, and operating pressure were shown to be the three factors that had the most impact on the permeate flux and Congo red dye rejection rate. The factors that affect the PES/PRS MMMs' CR dye rejection percentage regarding the rejection percentage and permeate flux were examined. A mathematical model was developed to determine the rejection percentage and permeate flux. The findings indicate that the key factors had a combined impact on the rejection percentage and permeate flux. According to the optimized variables, with a PRS of 64.43 mg and a CR concentration of 210 ppm, the permeate flux and rejection percentage of PES/PRS MMMs were 48.44 kg m^−2^ h^−1^ and 99.87%, respectively.

## Author contributions

Yusur Yahia: investigation and writing – original draft. Khalid T. Rashid: conceptualization, methodology, validation, writing – review & editing, and supervision. Mohammed Ahmed Shehab: investigation, visualization, and writing – review & editing. Adnan A. Abdul Razak: writing – original draft preparation. Maryam Y. Ghadhban: visualization and writing – review & editing. Munaf Al-lami, Mohammed A. Taher Al-Mayyahi: investigation, validation, and writing – review & editing. Mohammed A. Salih: validation and formal analysis. Haidar Hasan Mohammed: writing – review & editing. Alhafadhi Mahmood: formal analysis.

## Conflicts of interest

The authors declare no conflict of interest.

## Data Availability

The data supporting this article has been included within the article.
